# FAP as a Clinical Biomarker: FAP+ CAFs Orchestrate an Immunosuppressive Stem Cell Niche via the COL1A1/2‐CD44 Axis in Colon Cancer

**DOI:** 10.1155/sci/4678145

**Published:** 2026-08-06

**Authors:** Qian Ding, Qingyan Lu

**Affiliations:** ^1^ Department of Clinical Laboratory, Nanjing BenQ Medical Center, Nanjing, Jiangsu, China; ^2^ Department of Clinical Laboratory, Xuzhou Central Hospital, Xuzhou Clinical School of Xuzhou Medical University, Xuzhou, Jiangsu, China, xzch.cn

**Keywords:** cancer-associated fibroblasts, cancer stem cells, CD44, colon cancer, immunosuppressive microenvironment, fibroblast activation protein

## Abstract

**Background:**

Colon cancer (CC) remains a leading cause of cancer‐related mortality worldwide, driven largely by the complex interactions within the tumor microenvironment (TME). Fibroblast activation protein (FAP) is highly expressed in cancer‐associated fibroblasts (CAFs) and is associated with poor prognosis, yet its role in coordinating immune evasion and cancer stemness remains to be fully elucidated.

**Methods:**

We integrated multiomics data from TCGA and GEO databases, utilizing bulk RNA‐seq and single‐cell RNA‐seq (scRNA‐seq) analyses. Findings were validated via tissue microarray (TMA) immunohistochemistry (IHC). We further employed cell–cell communication analysis, pseudotime trajectory modeling, and the Connectivity Map (CMap) for drug sensitivity prediction and molecular docking.

**Results:**

FAP was significantly upregulated in CC tissues and correlated with advanced clinical stages. scRNA‐seq confirmed that FAP is predominantly expressed in CAFs. Functional analysis revealed that FAP‐high tumors are enriched in extracellular matrix (ECM) remodeling and immunosuppressive pathways. Cell–cell communication analysis identified that FAP+ CAFs interact with T cells and cancer stem cells (CSCs) primarily through the COL1A1/2‐CD44 axis. Specifically, FAP+ CAFs promote the differentiation of naive T cells into regulatory T cells (Tregs) and are positively correlated with various stemness markers, including CD44, ABCG2, and BMI1. Based on these findings, we established a 14‐gene prognostic risk model with robust predictive accuracy (area under the curve [AUC] > 0.64) and identified AS604850 and LY364947 as potential therapeutic agents.

**Conclusion:**

Our study demonstrates that FAP+ CAFs orchestrate a dual‐functional “immunosuppressive stem cell niche” via the COL1A1/2‐CD44 signaling axis. Targeting this FAP‐driven niche provides a promising strategy for overcoming immunotherapy resistance and improving clinical outcomes in CC.

## 1. Introduction

Colon cancer (CC) is a frequently lethal disease affecting the digestive system. Over the past few years, it has remained the third most common malignancy in terms of both incidence and mortality, impacting millions of people worldwide [1, 2]. Therefore, it is crucial to gain new insights into the role of tumor microenvironment (TME) cells and the molecular mechanisms that were involved in the development and malignant progression of CC.

Fibroblast activation protein (FAP) is a transmembrane glycoprotein that is sparsely present in healthy adult tissue but exhibits primarily significant expression in cancer‐associated fibroblasts (CAFs), which are found in the stroma of most epithelial tumors and are known to play a role in the growth and progression of tumors [3–5]. In CC, CAFs were significantly associated with poor prognosis in CC [6]. The release of paracrine active tumorigenic secretomes from CAFs played an important role in the process of CC metastasis [7]. Upregulation of FAP in fibroblasts was also reported to regulate CC cell motility, macrophage infiltration, and M2 polarization [8]. Moreover, CAFs could induce an immune‐excluded microenvironment, in which the proportion of CD8‐positive T cells is very low [9]. The tumorigenic characteristics of CAFs and their scarcity in most healthy tissues position FAP as a promising target for antibody conjugates and for the advancement of radiopharmaceuticals with CC applications [10]. For example, 68Ga‐labeled FAP inhibitor‐04 (68Ga‐FAPI‐04) PET/CT imaging could be used to monitor the response of metastatic colorectal cancer to TGF‐β inhibition and immunotherapy combination therapy, indicating the powerful role of 68Ga FAPI PET/CT imaging in evaluating tumor immunity and immunotherapy response in metastatic colorectal cancer [11].

The formation of an immunosuppressive state within the TME is a crucial process in tumor progression [12]. Regulatory T cells (Tregs) are a crucial type of suppressive immune cells that can inhibit the function of effector T cells through various modes of action, including cell surface inhibitors, inhibitory cytokines, or direct cytotoxicity [13–16]. Emerging preclinical data show that inducing depletion of Tregs or inhibiting their function could promote tumor regression [17–19]. In CC, the inhibition of Treg immunosuppression was found to partially restore CD8+ T cell and NK cell–mediated antitumor immunity and to prevent postoperative lung metastasis of CC cells [20]. Furthermore, Tregs that produce IL‐10 can enhance PD‐L1 expression in monocytes, subsequently decreasing CD8+ T cell infiltration and associated antitumor immunity in the context of liver metastases from colorectal cancer [21]. Moreover, apoptotic Tregs might exhibit increased immunosuppressive properties, resulting in immunotherapy that is less effective due to the blocking of immunosuppressive pathways. Nonetheless, the safe depletion of Tregs continues to pose a significant challenge. Additionally, cancer cells can promote tumor immune escape by infiltrating the TME and recruiting suppressive immune cell populations that inhibit the effector functions of various immune cells [22].

In addition to immune evasion, tumor progression is heavily dependent on a subpopulation of cells known as cancer stem cells (CSCs), which possess formidable capacities for self‐renewal, multilineage differentiation, and therapeutic resistance [23]. In CC, the maintenance of CSC stemness does not occur in isolation but requires continuous supportive signals from a specialized surrounding structure termed the “stem cell niche.” CAFs are increasingly recognized as critical architects of this niche, providing physical scaffolds and paracrine factors that nurture CSCs [24]. Notably, CD44, a classical transmembrane glycoprotein, serves not only as a universal marker for colon CSCs [25] but also acts as a primary receptor for extracellular matrix (ECM) components, including collagens and hyaluronic acid [26]. Despite its known role in mediating cell‐matrix interactions, whether the CAF‐derived collagen matrix regulates both immune cell differentiation and CSC maintenance simultaneously through CD44 remains largely elusive.

Therefore, we hypothesize that FAP+ CAFs may orchestrate a dual‐functional microenvironment, acting as both an immunosuppressive shield and a stem cell niche. In this study, we integrated multiomics bulk RNA‐seq data, single‐cell RNA‐seq (scRNA‐seq) analysis, and tissue microarray (TMA) validation to comprehensively decode the role of FAP in CC. We aimed to elucidate how FAP+ CAFs interact with T cells and CSCs via the COL1A1/2‐CD44 signaling axis, ultimately providing novel insights into the specific mechanisms of immune evasion and identifying potential therapeutic targets for overcoming treatment resistance in CC.

## 2. Materials and Methods

### 2.1. Data Acquisition and Preprocessing

We acquired RNA‐seq data for 23 normal specimens and 275 CC specimens from the TCGA database (https://portal.gdc.cancer.gov). Additionally, we also downloaded two datasets from the GEO database (https://www.ncbi.nlm.nih.gov/geo/) to analyze tissues from control individuals and CC patients: the GSE17536 cohort, which comprises 177 CC cases, and the GSE39582 cohort, which includes 433 test data cases of CC and 19 normal cases and 123 validation data cases of CC. The TCGA, GSE17536, and GSE39582 datasets were merged, and batch effects were removed using the sva package, which was visualized into the principal component analysis (PCA). Moreover, single‐cell data relating to CC were downloaded from the GSE166555 dataset within the GEO database, preserving 13 tumor samples and 12 normal samples for future analyses.

### 2.2. Identification and Exploration of FAP in CC

Using bulk RNA, we demonstrate the differences in FAP between tumor and normal tissues. The relationship between FAP and tumor grade is shown through box plots.

The quality of the scRNA‐seq data from the GSE166555 dataset was assessed and rigorously filtered using the “Seurat” package in R. Specifically, to exclude low‐quality cells, empty droplets, and potential multiplets, cells were retained for downstream analysis only if they met the following strict quality control (QC) thresholds: (1) the number of expressed genes per cell (nFeature_RNA) was between 500 and 10,000; (2) the total number of unique molecular identifiers (nCount_RNA) was between 1000 and 100,000; (3) the percentage of mitochondrial gene expression was less than 35%; and (4) the percentage of hemoglobin gene expression was less than 5%. The filtered scRNA‐seq data were then normalized through the “LogNormalize” method with a scale factor of 10,000, followed by the identification of the top 2000 highly variable genes using the “vst” selection method. Subsequently, the batch effects were corrected utilizing the “harmony” package. We then utilized the “harmony” package to correct for batch effects among the samples. To cluster the cells, we employed the “Find Neighbors” and “Find Clusters” functions, applying a resolution level of 0.8, and subsequently created a UMAP plot to visualize the data dimensionality of these clusters. Afterward, we annotated the cell clusters by referring to marker genes provided on the official CellMarker platform (http://xteam.xbio.top/CellMarker/). Violin plots and UMAP plots indicate the distribution of FAP in various cells. It is found that there are significant differences in expression in fibroblasts.

### 2.3. TMA and Immunohistochemistry (IHC) Staining

The CC TMA construction, IHC staining, and subsequent pathological evaluation were conducted by Shanghai Outdo Biotech Company (Shanghai, China). Briefly, the TMA sections were deparaffinized, rehydrated, and subjected to antigen retrieval, following standard protocols. The sections were then incubated with a primary antibody against FAP (Affinity, AF5344, 1:500). The staining signal was visualized using a standard DAB substrate kit, and the cell nuclei were counterstained with hematoxylin.

The IHC staining results were independently evaluated by two experienced pathologists in a double‐blinded manner. The expression level of FAP was assessed using a standard semiquantitative scoring system based on both the staining intensity and the percentage of positively stained cells in the cytoplasm. The staining intensity was graded as a continuous variable reflecting weak‐to‐strong staining, and the positive rate was recorded as the actual percentage of positively stained cells (ranging from 0% to 100%). The final IHC expression score was calculated by multiplying the staining intensity score by the positive rate percentage. Based on the median FAP expression score, the samples were subsequently stratified into FAP‐high and FAP‐low expression groups for clinical correlation and survival analysis.

### 2.4. Functional Enrichment Analyses

Genes associated with FAP expression using correlation analysis are identified, applying a filtering criterion of *p*  < 0.01. Subsequently, the correlations among the top 20 identified genes are presented. We constructed a protein–protein interaction (PPI) network utilizing the STRING database. To identify genes with similar functions and predict their biological roles, we employed the GeneMANIA database.

Based on the median expression levels of the identified FAP genes, we categorized the CC samples into two distinct groups. To evaluate the differential expression of all genes between these classifications, we employed the R package “limma.” Differential expression analysis was conducted, leading to the identification of 143 differentially expressed genes (DEGs) that met the criteria of |log2 FC| > 1 and an adjusted *p*‐value (FDR) < 0.05. These DEGs were subsequently subjected to enrichment analysis. Following this, we performed gene set enrichment analysis (GSEA) on the complete set of genes using the R package “ClusterProfiler” with a significance threshold established at *p*  < 0.05.

### 2.5. TME Analysis

Initially, we employed the ESTIMATE algorithm to assess the stromal and immune scores in cohorts with high and low expressions of FAP. Additionally, we conducted single‐sample GSEA (ssGSEA) to quantify the presence of 16 types of tumor‐infiltrating immune cells within each sample. Furthermore, the tumor immune dysfunction and exclusion (TIDE) tool (http://tide.dfci.harvard.edu/) was utilized to evaluate the potential efficacy of immune checkpoint blockades (ICBs) across various risk categories. To analyze the differences between these cohorts, we applied the Wilcoxon test. Concurrently, we performed a differential analysis of immune checkpoints between the high and low FAP expression groups, focusing on PDCD1, PDCD1LG2, CTLA4, CD80, CD86, and CD274.

### 2.6. Analysis of Cell–Cell Communication and Construction of Pseudotime Trajectory

We extracted single cells among fibroblasts and tumor cells for further dimensionality reduction clustering. We then further annotated T cells by referring to marker genes provided on the official CellMarker platform (http://xteam.xbio.top/CellMarker/). Employing the R package “CellChat,” we delved into the intercellular communication within the fibroblasts and T cell ligand, receptor pairs that contribute to cross‐talk between distinct cell types.

Single‐cell pseudotime analysis was established by Monocle 2. We selected a set of ordering genes that were expressed in at least 10% of all cells. “DDRTree” was applied to reduce the dimensionality of high‐dimensional data, which helps determine the trajectory of T cells. “DifferentialGeneTest” described DEG variations over pseudotime during T cell transformation. A heat map displaying the top 100 genes associated with T cell differentiation is presented. Additionally, we performed enrichment analysis to illustrate the functions related to Treg differentiation. To elucidate the regulatory relationship between CD44 and genes involved in Treg differentiation, we utilized the STRING database to construct a PPI network.

### 2.7. Drug Prediction and Molecular Docking

Connectivity Map (CMap) is an online resource with the potential to discover new drugs and stimulate novel hypotheses regarding connections among diseases, genes, and therapeutic drugs (http://clue.io). We input 137 upregulated genes associated with FAP into the CMap official website and analyzed the results obtained. Initially, a model diagram was employed to illustrate the small molecules that interact with pathways related to FAP activation. Subsequently, a heat map was utilized to display the relevant drug scores, where a lower score indicates a more favorable effect of the small‐molecule drug. Furthermore, for semiflexible docking studies, AutoDock Vina v1.1.2 software was used to examine the interactions involving AS604850, LY364947, and FAP.

### 2.8. Construction of Regression Model

Univariate Cox survival analysis was employed to identify FAP‐related differential genes. A *p*‐value of less than 0.05 was deemed significant, indicating a relationship with the survival of CC patients. The Least Absolute Shrinkage and Selection Operator (LASSO)–penalized Cox regression was applied to the overlapping genes to confidently select the most crucial genes for CC prognosis, thereby constructing a risk regression model. The survival differences between high‐ and low‐risk groups are illustrated using receiver operating characteristic (ROC) curves, survival curves, and survival scatter plots.

### 2.9. Statistical Analysis

Statistical evaluation and bioinformatics analyses were conducted using R software (version 4.3.3). To ensure the reproducibility of our computational analyses, the specific versions of all critical R packages utilized in this study are explicitly stated as follows: For scRNA‐seq data processing, QC, dimensionality reduction, and clustering, the “Seurat” package (version 5.0.1) was employed. Batch effects were corrected using the “harmony” package (version 1.2.0). Cell–cell communication analysis was comprehensively performed utilizing the “CellChat” package (version 1.6.1). For the single‐cell pseudotime trajectory inference and branched expression analysis, the “monocle 2” package (version 2.30.0) was applied. The LASSO‐penalized Cox regression analysis for selecting optimal prognostic genes and calculating risk scores was implemented using the glmnet package (version 4.1‐8). Other standard analytical and visualization packages, including “survival,” “survminer,” “ggplot2,” and “limma,” were used with their default parameters corresponding to the aforementioned R environment. The two‐tailed paired Wilcoxon signed–rank test was implemented to evaluate the differences in FAP IHC expression scores between paired CC tissues and adjacent normal tissues. The Pearson or Spearman correlation coefficient was utilized depending on data distribution. A survival analysis was carried out using survival data from TCGA and GEO. The survival and survminer R packages, with default parameters, were used to assess the levels of gene expression.

## 3. Results

### 3.1. Identification of FAP Expression and Its Clinical Relevance in CC

Firstly, we expanded the sample size by integrating three datasets to analyze the expression of FAP in CC. A total of 1062 tissue samples were collected, including 285 CC patient tissue samples and 25 normal tissue samples in the TCGA‐COAD dataset, 177 CC patient tissue samples in the GSE17536 dataset, 433 CC patient tissue samples in the GSE39582 dataset, 19 normal tissue samples in the test dataset, and an additional 123 CC patient tissue samples in the validation dataset. PCA shows that the datasets are far apart, indicating significant differences and clear batch effects (Figure 1A). We further utilize the sva package to remove batch effects from the samples and integrate the three datasets. Performing PCA again suggests a significant similarity between samples for further analysis (Figure 1B). Furthermore, the analysis of the integrated dataset results shows that FAP is significantly upregulated in CC tissues compared to normal tissues (Figure 1C). We also found a significant correlation between the expression of the FAP gene and clinical stage (Figure 1D). Furthermore, to assess the prognostic value of FAP, we performed Kaplan–Meier survival analysis. The results indicated that patients with high FAP expression exhibited a trend towards worse progression‐free survival (PFS) compared to those with low FAP expression (hazard ratio = 1.13, 95% CI: 1.03–1.24, stratified log‐rank *p* = 0.057) (Figure 1E). To further validate the differential expression of FAP at the protein level, we performed IHC staining on a TMA containing 85 paired CC tissues and adjacent normal tissues, along with five metastatic CC specimens (Figure 1F). Consistent with our transcriptional analysis from the public databases, the paired sample analysis demonstrated that the FAP protein levels were significantly elevated in CC tissues compared to their corresponding adjacent normal tissues (*p*  < 0.0001) (Figure 1G). These results robustly confirm the significant upregulation of FAP in CC.

Figure 1Identification of FAP expression and its clinical relevance in colon cancer. (A, B) Principal component analysis (PCA) of the integrated datasets (TCGA‐COAD, GSE17536, and GSE39582, *n* = 1062) before (A) and after (B) batch effect removal. (C) Violin plot demonstrating the significant upregulation of FAP expression in colon cancer tissues compared to normal tissues in the integrated dataset. (D) Violin plot illustrating the positive correlation between FAP expression levels and advanced clinical stages (Stages I to IV). (E) Kaplan–Meier survival curve evaluating progression‐free survival based on high versus low FAP expression. (F) Representative immunohistochemistry (IHC) images of the tissue microarray containing colon cancer tissues, adjacent normal tissues, and metastatic specimens. (G) Comparison of FAP IHC expression scores between paired colon cancer tissues (tumor) and adjacent normal tissues (normal). *p*  < 0.0001 by the two‐tailed paired Wilcoxon signed–rank test.

**Figure:**
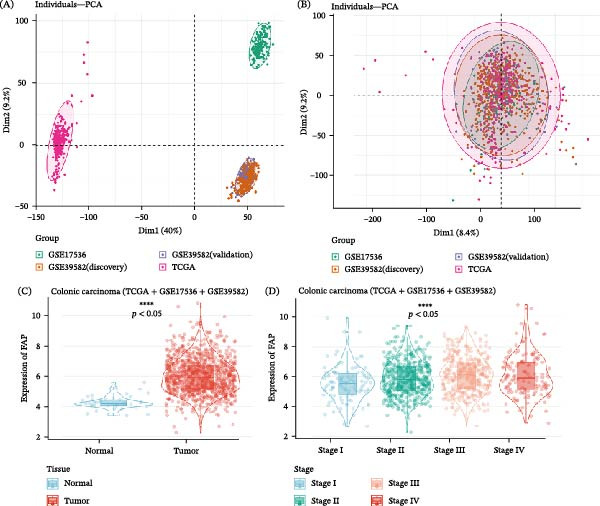

**Figure:**
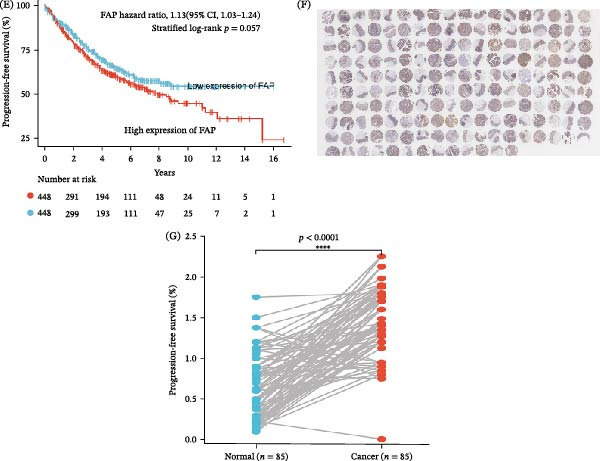

### 3.2. scRNA‐Seq Analysis of FAP Expression

**Figure 2:**
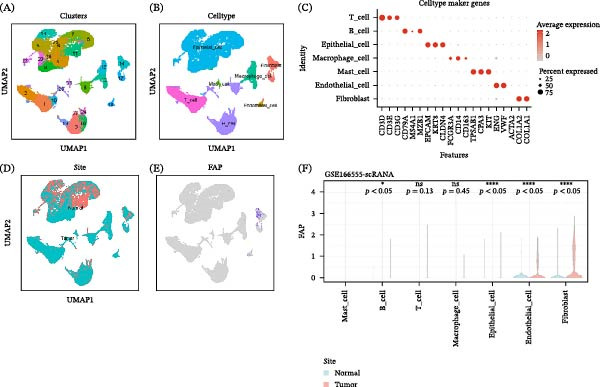
Single‐cell RNA sequencing (scRNA‐seq) analysis of FAP expression. (A) UMAP plot displaying the dimensionality reduction and clustering of 27 distinct cell clusters from the GSE166555 dataset. (B) UMAP plot of the seven annotated major cell types within the tumor microenvironment. (C) Bubble plot illustrating the average expression and percentage of specific marker genes used for cell type identification. (D) UMAP plot showing the distribution of cells derived from normal and tumor sites. (E) UMAP plot highlighting the specific distribution of FAP gene expression, predominantly located in the fibroblast cluster. (F) Violin plots comparing FAP expression levels across different cell types between normal and tumor tissues.

We used the GSE166555 dataset from the GEO database to analyze the expression of the FAP gene in different cell types. The scRNA‐seq data were processed and divided into 27 clusters using UMAP dimension reduction (Figure 2A). According to the CellMarker official website, single‐cell data are annotated and divided into seven major cell types (Figure 2B). The expression bubble plot of marker genes in cells can be found in Figure 2C. The grouping of cells from tumor and normal samples is shown in Figure 2D. Figure 2E displays the distribution of FAP gene expression across these seven cell populations. The results demonstrated that FAP is predominantly expressed in fibroblasts. Moreover, we compared the expression levels of FAP in different cell populations stratified by the sample source. Figure 2F shows violin plots highlighting statistically significant expression of FAP in B cells, epithelial cells, endothelial cells, and fibroblasts. These findings indicated that the FAP gene is mainly expressed in fibroblasts, and its expression levels may be influenced by the degree of malignancy.

### 3.3. Identification and Functional Enrichment of FAP‐Correlated Genes

To explore the genes associated with FAP expression in CC, we performed Spearman correlation analysis using the integrated sample dataset. The analysis identified the top genes most closely and positively correlated with FAP expression, with CTHRC1 and SULF1 showing the highest correlation coefficients (Cor > 0.85) (Figure 3A). Subsequently, to elucidate the biological functions of these highly correlated genes, we performed functional enrichment analysis. A Sankey diagram was generated to visualize the mapping between these key genes and their significantly enriched Gene Ontology (GO) terms (Figure 3B). The results revealed a strong enrichment in matrix‐related functions, including “collagen‐containing extracellular matrix,” “extracellular matrix structural constituent,” and “extracellular matrix organization.” Furthermore, we constructed a PPI network utilizing the STRING database to present the complex direct and indirect interactions among FAP and these correlated genes (Figure 3C).

Figure 3Identification and functional enrichment of FAP‐correlated genes. (A) Lollipop chart showing the top 20 genes most closely and positively correlated with FAP expression (Spearman correlation). (B) Sankey diagram visualizing the mapping between highly correlated genes and their significantly enriched Gene Ontology (GO) terms, primarily focusing on extracellular matrix organization. (C) Protein–protein interaction (PPI) network of FAP and its correlated genes constructed using the STRING database.

**Figure:**
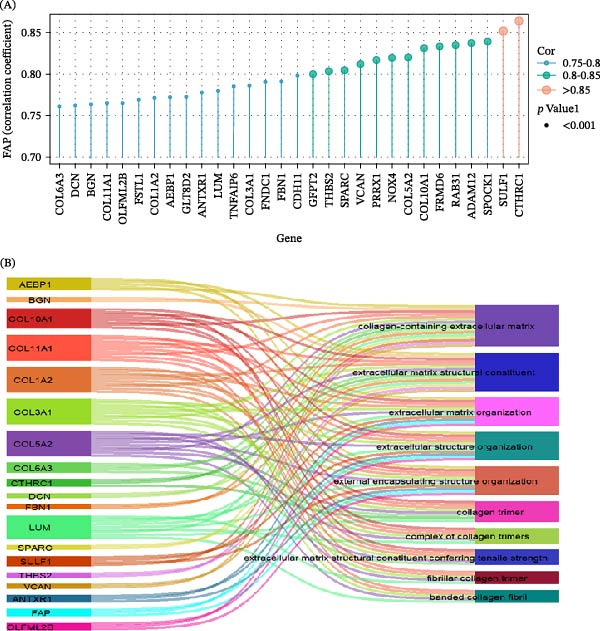

**Figure:**
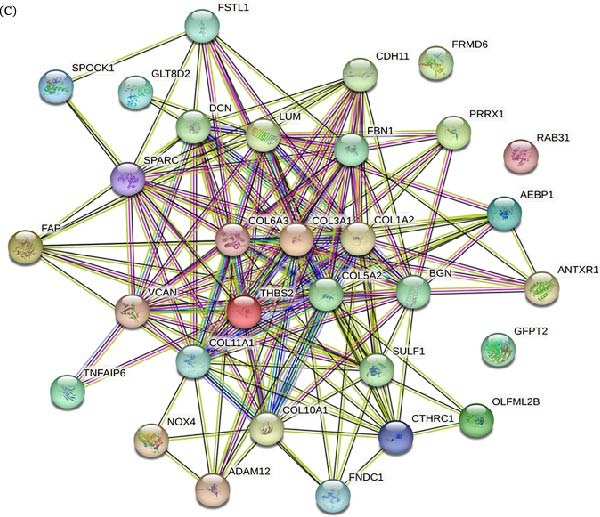

### 3.4. Transcriptomic Landscape and Signaling Pathway Activation Regulated by FAP

To further investigate the downstream mechanisms associated with FAP, we divided the integrated CC dataset into high‐ and low‐expression groups based on the median FAP expression level. Differential expression analysis was performed using the “limma” package (filtering criteria: adjusted *p*‐value (FDR) < 0.05 and |log2FC| > 1). The volcano plot revealed 137 significantly upregulated genes and six downregulated genes in the FAP‐high group (Figure 4A). Subsequently, GO and KEGG pathway enrichment analyses were conducted on these DEGs. The results demonstrated that these DEGs were primarily enriched in biological processes and cellular components such as ECM organization, extracellular structure organization, and collagen‐containing ECM. The KEGG analysis indicated significant enrichment in ECM–receptor interaction, protein digestion and absorption, focal adhesion, and chemokine signaling pathways (Figure 4B).

Figure 4Transcriptomic landscape and signaling pathway activation regulated by FAP. (A) Volcano plot displaying the differentially expressed genes (DEGs) between the FAP‐high and FAP‐low expression groups (|log2FC| > 1, *p*  < 0.05). (B) Bar charts detailing the Gene Ontology (GO) and KEGG pathway enrichment analyses of the DEGs. (C) Gene set enrichment analysis (GSEA) plots highlighting significant oncogenic and immune‐related signaling pathways (including ECM–receptor interaction, Toll‐like receptor, NF‐κB, PI3K‐Akt, and PD‐L1 expression) positively enriched in the FAP‐high group.

**Figure:**
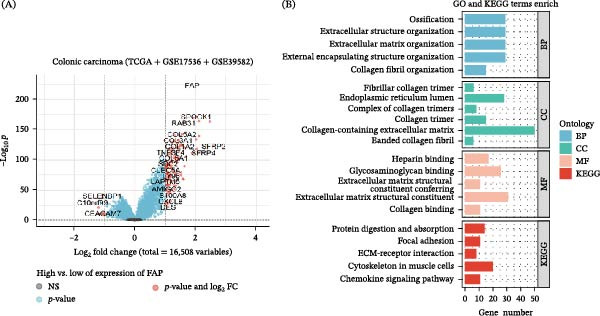

**Figure:**
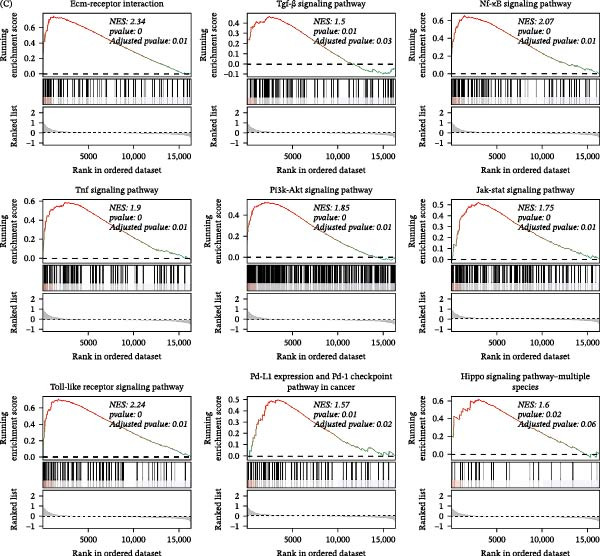

Moreover, GSEA was performed to identify the key signaling pathways activated in the FAP‐high group. The GSEA results revealed that high FAP expression was significantly and positively enriched in multiple critical tumor progression and immune‐related pathways, including the ECM–receptor interaction (normalized enrichment score [NES] = 2.34, *p*  < 0.001), Toll‐like receptor signaling pathway (NES = 2.24, *p*  < 0.001), NF‐κB signaling pathway (NES = 2.07, *p*  < 0.001), TNF signaling pathway (NES = 1.90, *p*  < 0.001), PI3K‐Akt signaling pathway (NES = 1.85, *p*  < 0.001), JAK‐STAT signaling pathway (NES = 1.75, *p*  < 0.001), Hippo signaling pathway (NES = 1.60, *p* = 0.02), TGF‐β signaling pathway (NES = 1.50, *p* = 0.01), and PD‐L1 expression and PD‐1 checkpoint pathway in cancer (NES = 1.57, *p* = 0.01) (Figure 4C). These findings strongly suggest that FAP promotes CC progression by extensively remodeling the ECM and regulating crucial immune and inflammatory signaling pathways.

### 3.5. Immune Landscape and Immunotherapeutic Response Analysis Associated With FAP

To explore the association between FAP and the tumor immune microenvironment (TME), we employed the ESTIMATE algorithm to evaluate the TME pattern in the integrated CC dataset. The results indicated that the FAP‐high expression group exhibited significantly higher ESTIMATEScore, ImmuneScore, and StromalScore compared to the FAP‐low group (*p*  < 0.05) (Figure 5A). The elevated stromal and immune scores suggest that FAP is closely involved in the complex interplay between matrix remodeling and immune cell infiltration.

Figure 5Immune landscape and immunotherapeutic response analysis associated with FAP. (A) Violin plots comparing the ESTIMATEScore, ImmuneScore, and StromalScore between the FAP‐high and FAP‐low groups. (B) Box plots showing the differences in the abundance of 16 infiltrating immune cell types between risk groups via ssGSEA. (C) Scatter plot demonstrating a significant positive correlation between FAP expression and the regulatory T cell (Treg) score. (D) Violin plots evaluating immunotherapeutic efficacy metrics, including dysfunction, exclusion, MSI.Expr.Sig, and overall TIDE scores across FAP expression groups. (E) Correlation heatmap mapping the relationship between FAP and various critical immune checkpoint molecules.

**Figure:**
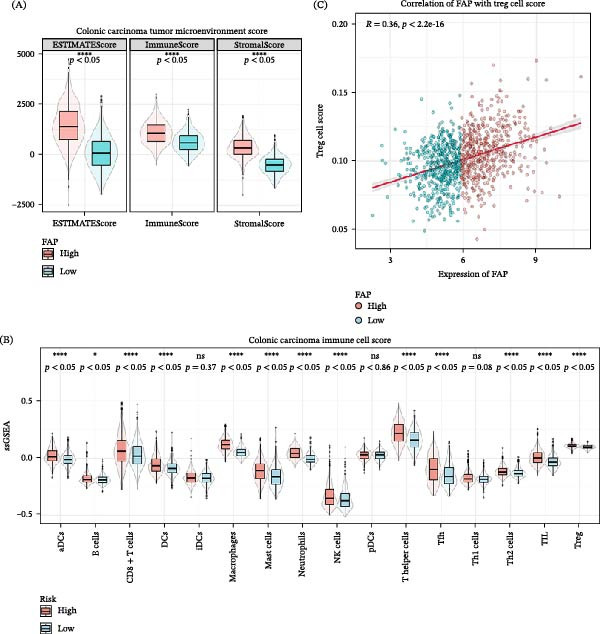

**Figure:**
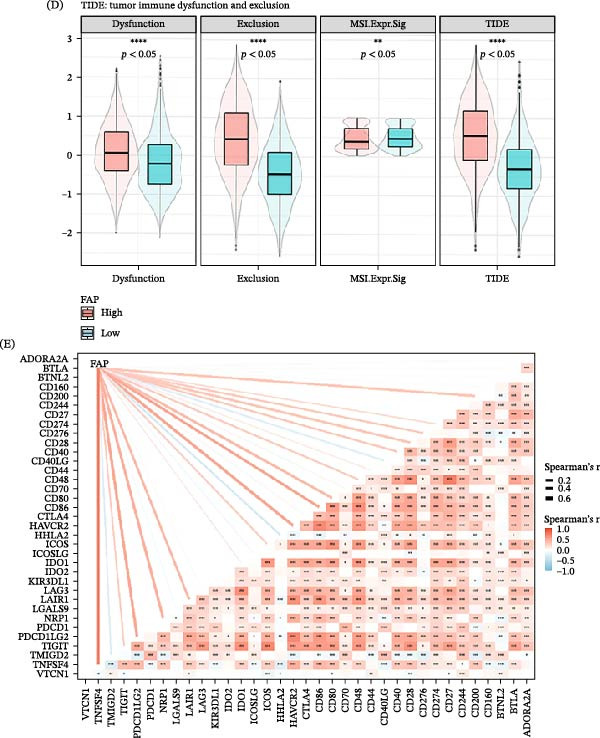

To further detail the specific immune cell populations, we performed ssGSEA. The analysis demonstrated significant differences in the abundance of various immune cells between the two risk groups, notably showing an elevated infiltration of immunosuppressive cells such as Tregs and macrophages in the high‐risk group (Figure 5B). Given the critical role of Tregs in mediating tumor immune escape, we specifically investigated their relationship with FAP. A significant positive correlation was observed between FAP expression and the Treg cell score (*R* = 0.36, *p* < 2.2 *e*−16) (Figure 5C), suggesting that FAP may promote the recruitment or generation of Tregs in the TME.

Furthermore, we evaluated the potential efficacy of immunotherapy using the TIDE algorithm. The FAP‐high group demonstrated significantly higher TIDE, T cell dysfunction, and T cell exclusion scores, along with a lower microsatellite instability (MSI.Expr.Sig) score (Figure 5D). These metrics strongly imply that high FAP expression mediates an immune‐excluded or exhausted microenvironment, rendering these patients more prone to ICB resistance. Consistently, our correlation analysis of immune checkpoint molecules revealed that FAP expression is robustly and positively correlated with a wide array of critical immune checkpoints, including CD274 (PD‐L1), CTLA4, PDCD1 (PD‐1), and HAVCR2 (TIM‐3) (Figure 5E). Collectively, these findings indicate that FAP extensively shapes an immunosuppressive microenvironment in CC, contributing to tumor immune evasion.

### 3.6. Cell–Cell Communication Between FAP+ Fibroblasts and T Cells

To deeply explore the association between FAP+ fibroblasts and Tregs, we extracted fibroblasts and T cells from the scRNA‐seq dataset for subclustering and dimensionality reduction. A total of 16 distinct cell clusters (Clusters 0–15) were identified (Figure 6A), which were broadly segregated into fibroblast and T cell lineages (Figure 6B). To annotate the specific cell identities, we evaluated the expression of canonical marker genes across the clusters (Figure 6C) and final cell populations (Figure 6D), referencing the CellMarker database. Cells highly expressing ACTA2 were identified as fibroblasts, which were further subdivided into FAP+ and FAP− fibroblasts based on FAP expression. For the T cell populations (expressing PTPRC), cells expressing CCR7 were defined as naive T cells, while those specifically expressing FOXP3 and IL2RA were annotated as Tregs. Furthermore, cells expressing CD4 but lacking FOXP3/IL2RA were classified as CD4+ T cells, those expressing CD8A as CD8+ T cells, and cells expressing PTPRC without other specific subset markers were defined as memory T cells. One cluster located on the periphery without distinct classical markers was denoted as unknown T cells. The final annotated landscape of these subsets is visualized in the UMAP plot (Figure 6E).

Figure 6Cell–cell communication between FAP+ fibroblasts and T cells. (A) UMAP plot showing the subclustering of extracted fibroblasts and T cells into 16 distinct clusters. (B) UMAP plot broadly segregating the fibroblast and T cell lineages. (C, D) Violin plots illustrating the expression levels of canonical marker genes across the 16 clusters (C) and the final annotated cell subpopulations (D). (E) UMAP plot visualizing the final annotated landscape of specific fibroblast (FAP+ and FAP−) and T cell subsets. Colors correspond to the specific cell identities as annotated in the plot. (F) Stacked bar chart comparing the proportional composition of these cell subsets in normal versus tumor tissues. (G) Bubble plot of ligand‐receptor communication networks, highlighting the prominent COL1A1/2‐CD44 signaling axes from FAP+ fibroblasts to naive T cells, CD4+ T cells, and Tregs.

**Figure:**
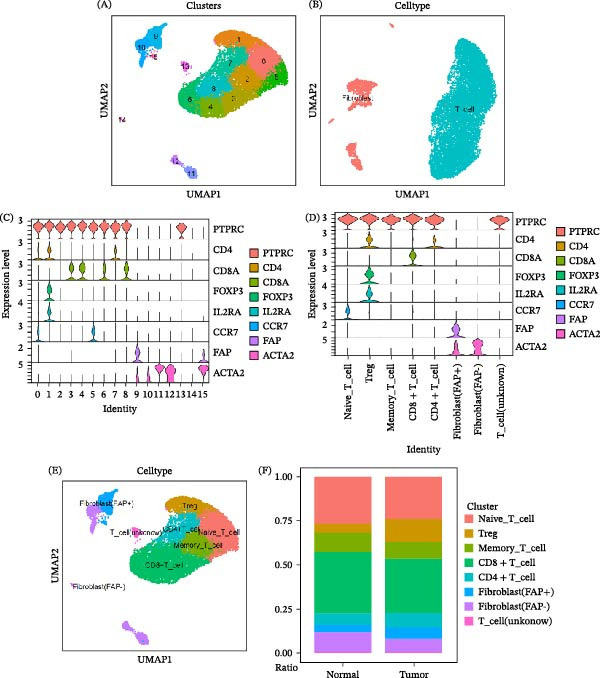

**Figure:**
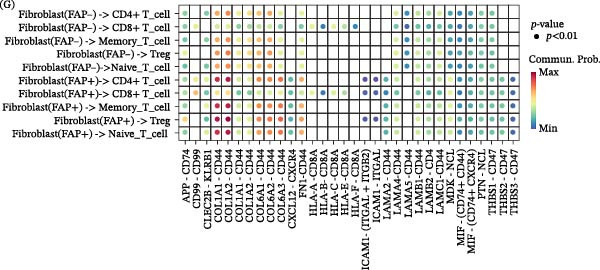

We subsequently compared the composition of these cell subsets across normal and tumor tissues. The proportional analysis revealed a notable expansion of both FAP+ fibroblasts and Tregs within the TME (Figure 6F). Given their concurrent enrichment and the significant correlation observed earlier, we hypothesized a functional interaction between FAP+ fibroblasts and Tregs. To characterize this crosstalk, we performed cell–cell communication analysis. The ligand‐receptor bubble plot demonstrated robust interaction networks between fibroblasts and T cells (Figure 6G). Strikingly, the communication directed from FAP+ fibroblasts to naive T cells, CD4+ T cells, and Tregs was heavily reliant on the COL1A1‐CD44 and COL1A2‐CD44 signaling axes. These findings indicate that FAP+ fibroblasts actively communicate with T cells via collagen‐CD44 interactions, potentially influencing T cell differentiation and shaping an immunosuppressive niche.

### 3.7. FAP+ Fibroblasts Promote Treg Differentiation via the CD44 Signaling Axis

To elucidate the underlying gene expression dynamics driving T cell fate decisions, we performed branched expression analysis modeling (BEAM). The resulting heatmap illustrated the top DEGs associated with differentiation, stratifying them into two major gene clusters with distinct expression patterns (Figure 7A). Subsequent functional enrichment analysis revealed that genes in Cluster 1 (highly expressed in the Treg‐associated branch) were significantly enriched in immune‐related functions, including T cell proliferation, regulation of T cell activation, and the T cell receptor signaling pathway (Figure 7B). Meanwhile, genes in Cluster 2 were mainly enriched in biological processes such as response to unfolded proteins and protein processing in the endoplasmic reticulum (ER) (Figure 7C).

Figure 7FAP+ fibroblasts promote Treg differentiation via the CD44 signaling axis. (A) Branched expression analysis modeling (BEAM) heatmap depicting the top differentially expressed genes driving T cell fate decisions over pseudotime, stratified into two major gene clusters. (B, C) GO and KEGG functional enrichment analyses for genes in Cluster 1 (B) and Cluster 2 (C). (D) Pseudotime trajectory plot generated by Monocle 2 identifying three distinct cell states. (E) Single‐cell trajectory colored by continuous pseudotime progression. (F) Trajectory map overlaying cell types, showing the developmental bifurcation from naive T cells into CD4+ T cells and Tregs. (G) PPI network centered on the CD44 receptor and key downstream Treg‐associated differentiation proteins (e.g., FOXP3, IL2RA, and CTLA4).

**Figure:**
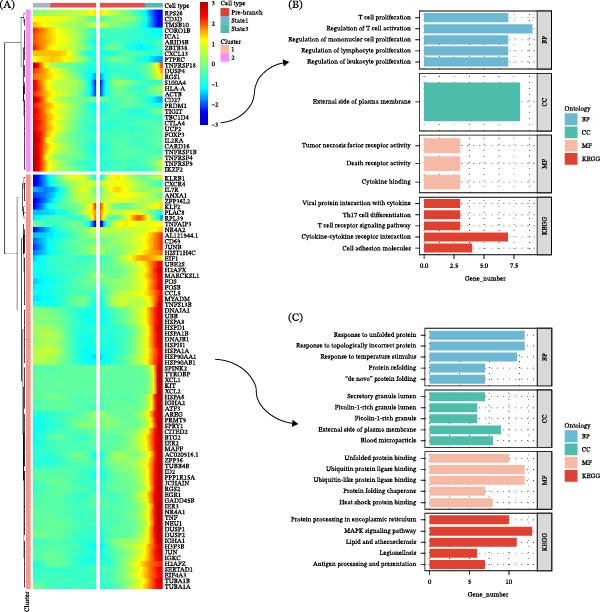

**Figure:**
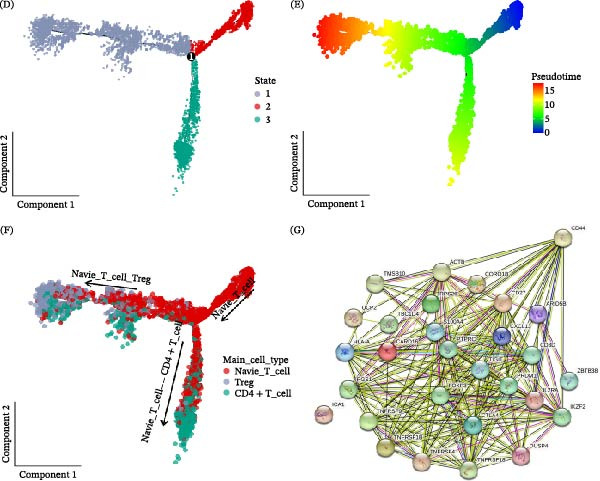

To visually reconstruct this developmental process at single‐cell resolution, we conducted cell trajectory analysis using the “monocle 2” R package. The pseudotime analysis identified a branched trajectory characterized by three distinct cell states (Figure 7D). By ordering cells along the pseudotime (Figure 7E) and mapping the defined cell types onto the trajectory, we observed a clear developmental progression: Naive T cells were predominantly located at the root of the trajectory and subsequently bifurcated into two distinct branches, with one branch differentiating into CD4+ T cells and the other directly leading to Tregs (Figure 7F).

Finally, to clarify the regulatory relationship between the identified receptor CD44 and Treg differentiation, we constructed a PPI network using the STRING database. The network demonstrated that CD44 profoundly and directly interacts with key proteins essential for Treg differentiation and function, including FOXP3, IL2RA, and CTLA4 (Figure 7G). Taken together, these results strongly support the mechanism that FAP+ fibroblasts orchestrate the differentiation of naive T cells into immunosuppressive Tregs via the CD44 signaling axis.

### 3.8. Correlation Between FAP Expression and Stemness Markers in CC

Given that CD44 is not only a crucial receptor mediating Treg differentiation but also a well‐established marker for CSCs [25], we further hypothesized that FAP expression might be closely associated with the maintenance of a stem cell niche. To validate this, we evaluated the correlation between FAP and a comprehensive panel of stemness‐related genes using the bulk RNA‐seq data from the TCGA CC cohort. Spearman correlation analysis revealed that FAP expression was significantly and positively correlated with several critical stemness and pluripotency markers, including CD44 (*R* = 0.187, *p*  < 0.001), ABCG2 (*R* = 0.308, *p*  < 0.001), BMI1 (*R* = 0.327, *p*  < 0.001), SOX2 (*R* = 0.199, *p*  < 0.001), and the intestinal stem cell marker LGR5 (*R* = 0.109, *p* = 0.017) (Figure 8A–E). Meanwhile, negative correlations were observed with EPHB2, POU5F1, and EPCAM (Figure 8F–H), whereas genes such as PROM1, KLF4, ALDH1A1, MYC, and NANOG exhibited no significant correlation (Figure 8I–M). Together with the single‐cell trajectory findings, these results strongly support our hypothesis that FAP+ CAFs orchestrate a dual‐functional microenvironment, serving as both an immunosuppressive and a stem‐like niche, potentially linked through the core CD44 signaling node.

Figure 8Correlation between FAP expression and stemness markers in colon cancer. (A–E) Scatter plots from TCGA bulk RNA‐seq data showing significant positive correlations between FAP expression and classical stemness/pluripotency markers, including *CD44* (A), *ABCG2* (B), *BMI1* (C), *SOX2* (D), and the intestinal stem cell marker *LGR5* (E). (F–H) Scatter plots demonstrating negative correlations between FAP and *EPHB2* (F), *POU5F1* (G), and *EPCAM* (H). (I–M) Scatter plots showing no significant correlation between FAP expression and *PROM1* (I), *KLF4* (J), *ALDH1A1* (K), *MYC* (L), and *NANOG* (M).

**Figure:**
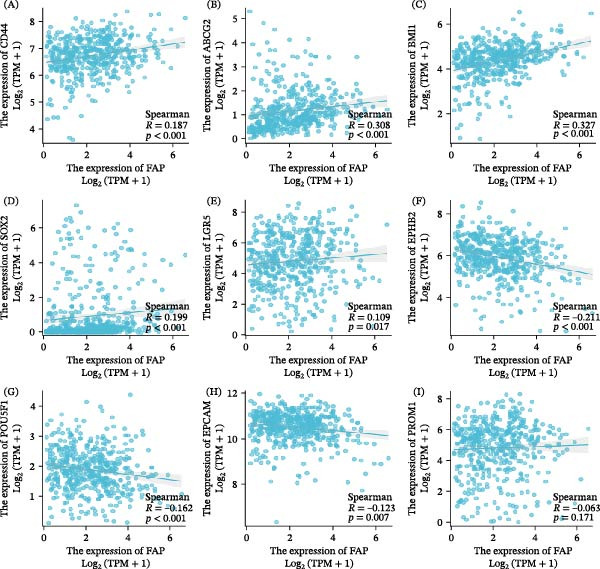

**Figure:**
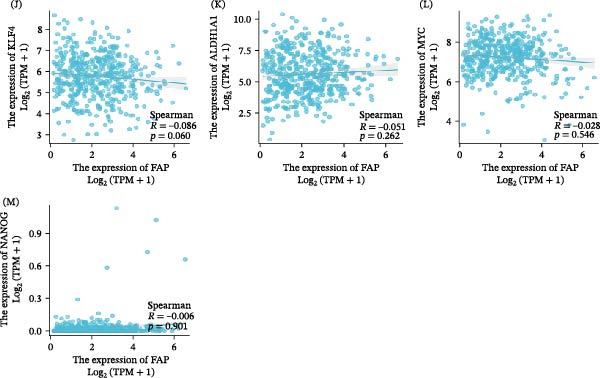

### 3.9. Drug Sensitivity Prediction and Molecular Docking

Based on our previous GSEA findings that high FAP expression is closely related to multiple oncogenic and inflammatory signaling pathways (such as PI3K‐Akt, JAK‐STAT, NF‐κB, TGF‐β, and TNF pathways), we sought to identify potential therapeutic agents. Using the CMap database, we screened for small‐molecule inhibitors targeting these specific mechanisms of action (Figure 9A). By evaluating the CMap scores, where a lower negative score indicates a higher potential to reverse the FAP‐associated gene expression signature, we identified several promising drug candidates (Figure 9B). Among them, two top‐ranked small‐molecule compounds with significant negative scores were selected for further investigation: AS604850 (a PI3K inhibitor) and LY364947 (a TGF‐β receptor inhibitor) (Figure 9C).

Figure 9Drug sensitivity prediction and molecular docking. (A) Classification of small‐molecule inhibitors targeting FAP‐associated mechanisms of action based on the Connectivity Map (CMap) database. (B) Heatmap of CMap scores identifying promising drug candidates capable of reversing the FAP‐associated gene expression signature. (C) 3D chemical structures of the selected therapeutic candidates (AS604850 and LY364947) and the FAP protein (Q12884). (D, E) Molecular docking simulations depicting the optimal spatial conformations and binding pockets of AS604850 (D) and LY364947 (E) with the FAP protein. (F) Bar chart comparing the robust binding energies of AS604850 and LY364947 to the FAP target.

**Figure:**
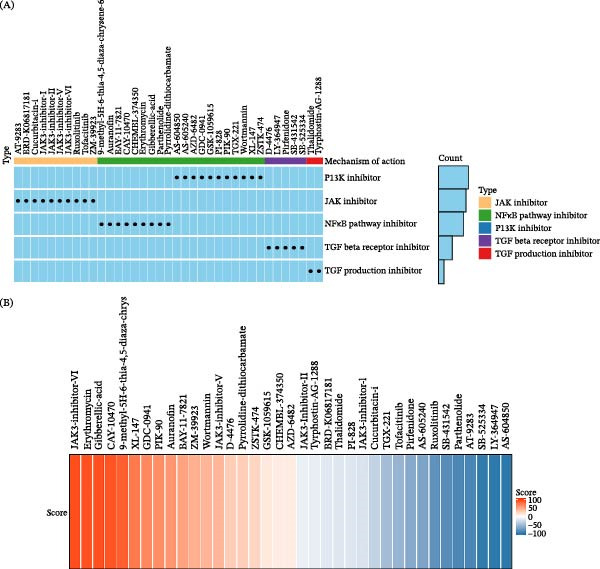

**Figure:**
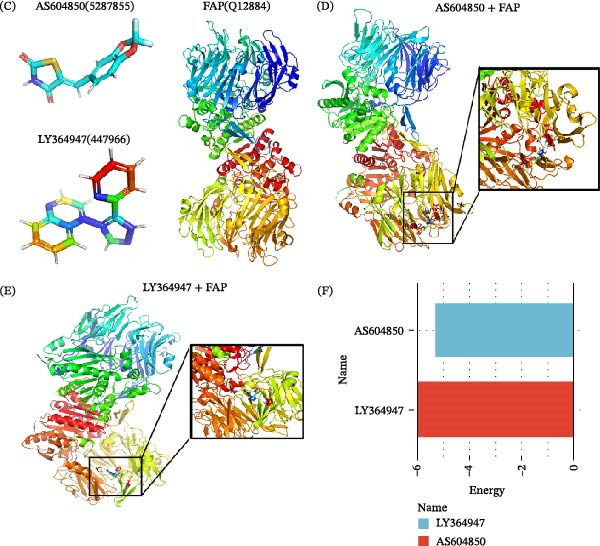

To further evaluate the direct interaction and binding affinity between these drugs and the FAP protein, we performed molecular docking simulations. The optimal spatial conformations and active binding pockets of AS604850 and LY364947 with the FAP protein (Q12884) are explicitly illustrated in Figure 9D,E, respectively. Both compounds exhibited robust binding affinities, with the binding energies recorded at approximately −5.5 kcal/mol for AS604850 and −6.0 kcal/mol for LY364947 (Figure 9F). These results theoretically suggest that AS604850 and LY364947 could serve as potential targeted therapeutics for intervening in FAP‐driven CC progression.

### 3.10. Establishment and Validation of a FAP‐Related Prognostic Risk Model

To evaluate the clinical prognostic value of FAP‐associated genes, we first identified 85 genes significantly associated with patient prognosis through univariate Cox survival analysis. To prevent overfitting and construct a robust prognostic signature, we applied the LASSO‐penalized Cox regression model to these candidates. Based on the optimal penalty parameter *λ* = 0.0803, 14 key genes (CCL8, SPARCL1, SFRP2, ARL4C, COMP, AOC3, LOX, SPP1, CD109, TMEM45A, OLFM4, C10orf99, SELENBP1, and CXCL10) were ultimately retained to establish the risk score model (Figure 10A). The respective nonzero coefficients of these 14 genes are detailed in Figure 10B.

Figure 10Establishment and validation of a FAP‐related prognostic risk model. (A) Cross‐validation plot for the LASSO‐penalized Cox regression model showing the partial likelihood deviance against the penalty parameter log(λ). (B) Bar chart detailing the specific nonzero coefficients for the 14 retained prognostic genes. (C) Time‐dependent ROC curves predicting the 1‐, 3‐, and 5‐year progression‐free survival (PFS) in the integrated dataset. (D) Kaplan–Meier survival analysis comparing PFS between the high‐risk and low‐risk groups (hazard ratio = 5.35, *p*  < 0.001). (E) Scatter plots visualizing the distribution of patient risk scores and their corresponding survival status. (F) Heatmap illustrating the distinct expression patterns of the 14 prognostic genes between the high‐risk and low‐risk groups.

**Figure:**
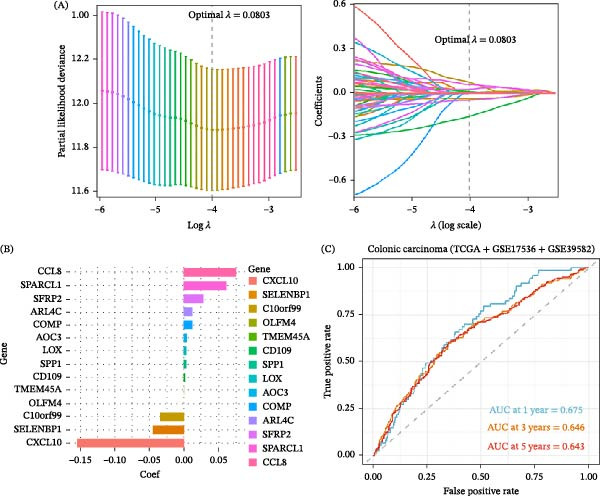

**Figure:**
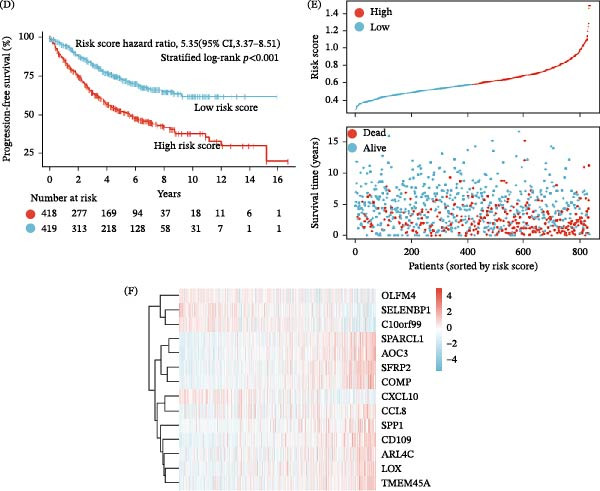

To evaluate the predictive capacity of our established model, we generated time‐dependent ROC curves. In the integrated dataset (comprising TCGA‐COAD, GSE17536, and GSE39582), the areas under the curve (AUCs) for predicting 1‐, 3‐, and 5‐year PFS were determined to be 0.675, 0.646, and 0.643, respectively (Figure 10C), indicating a favorable and stable predictive accuracy.

Subsequently, the patients in the integrated dataset were stratified into high‐risk and low‐risk groups based on the median risk score. Kaplan–Meier survival analysis demonstrated that patients in the high‐risk group exhibited a significantly worse PFS compared to those in the low‐risk group (hazard ratio = 5.35, *p*  < 0.001) (Figure 10D). Furthermore, the risk score distribution and survival status scatter plots consistently illustrated that higher risk scores were robustly associated with increased mortality rates and shorter survival times (Figure 10E). Finally, the distinct expression patterns of the 14 prognostic genes between the high‐ and low‐risk groups were visualized in a heatmap (Figure 10F).

## 4. Discussion

CC remains a global health challenge due to its high incidence and mortality rates. Emerging evidence underscores the critical role of TME in tumor progression and therapeutic resistance. In this study, we integrated multiomics analysis, including bulk RNA‐seq and scRNA‐seq, along with clinical TMA validation, to characterize the role of FAP in CC. We demonstrated that FAP is significantly upregulated in CC tissues and is primarily expressed in CAFs. More importantly, our findings reveal that FAP+ CAFs orchestrate a unique “immunosuppressive stem cell niche” by interacting with T cells and CSCs via the COL1A1/2‐CD44 signaling axis, thereby promoting immune evasion and tumor malignancy.

Consistent with previous reports, our analysis of the integrated TCGA and GEO datasets showed that the FAP expression is elevated in CC and correlates with advanced clinical stages. This was robustly validated at the protein level using a TMA containing 85 paired samples and five metastatic specimens, where FAP protein levels were markedly higher in cancerous and metastatic tissues than in adjacent normal tissues (Figure 1F,G). Functional enrichment analysis further revealed that FAP‐correlated genes, such as CTHRC1 and SULF1, are predominantly involved in ECM organization and collagen fibril assembly. These findings suggest that FAP‐expressing CAFs are the primary architects of the desmoplastic stroma in CC, providing a structural scaffold that facilitates tumor invasion and limits the infiltration of antitumor immune cells.

A hallmark of CC progression is the formation of an immunosuppressive microenvironment. Our TIDE analysis and ssGSEA results indicated that high FAP expression is associated with higher T cell exclusion and dysfunction scores, as well as increased infiltration of Tregs. Using scRNA‐seq and cell–cell communication analyses, we identified the COL1A1/2‐CD44 axis as the primary mediator of crosstalk between FAP+ CAFs and T cells. Pseudotime trajectory analysis further demonstrated that FAP+ fibroblasts promote the differentiation of naive CD4+ T cells into immunosuppressive Tregs. Interestingly, our branched expression analysis revealed that this fate decision process was accompanied by a significant enrichment of genes associated with the unfolded protein response (UPR) (Cluster 2). Biologically, this reflects the physiological adaptation of differentiating T cells to the tremendous biosynthetic demands required for rapid proliferation and the massive production of effector molecules and surface receptors. The acute surge in protein synthesis naturally induces ER stress. To cope with this stress and survive in the metabolically hostile TME, these differentiating cells upregulate the UPR, an essential driver for maintaining the metabolic fitness and suppressive function of Tregs. Mechanistically, the interaction between collagen ligands (COL1A1/2) and the CD44 receptor on T cells appears to stabilize key Treg‐associated proteins like FOXP3 and CTLA4 (Figure 7G). This mechanism explains how FAP+ CAFs actively “shield” tumor cells from immune surveillance by recruiting and inducing suppressive T cell populations.

Another pivotal finding of this study is the link between FAP+ CAFs and the tumor stem cell niche. CD44 is not only a receptor on T cells but also a hallmark of CSCs in the intestine [25, 26]. We observed that FAP expression is significantly and positively correlated with a suite of stemness markers, including CD44, ABCG2, BMI1, SOX2, and LGR5 (Figure 8A–E). These markers are essential for CSC self‐renewal, chemoresistance, and tumor initiation [27–29]. Through the high expression of collagenous ligands, FAP+ CAFs may interact with CD44 on CSCs to maintain their pluripotency and anchor them within a supportive niche. This dual role, promoting both immune suppression and stemness, positions FAP+ CAFs as a central regulator of the “immunosuppressive stem cell niche,” a concept that integrates two of the most critical drivers of CC malignancy.

The clinical utility of our findings was exemplified by the construction of a 14‐gene prognostic model, which showed robust predictive accuracy for 1‐, 3‐, and 5‐year survival (AUC > 0.64). Consistently, Zhang et al. also established a 20‐gene CAF‐related prognostic signature (CAFPS) in colorectal cancer and demonstrated that higher CAF risk scores were associated with poor prognosis and reduced immunotherapy response [30]. Furthermore, through CMap analysis and molecular docking, we identified AS604850 and LY364947 as potential therapeutic agents that target FAP‐related pathways with high binding affinity. While our study provides a comprehensive landscape of FAP in CC, further in vitro and in vivo experiments are required to validate the exact downstream signaling of the COL1A1/2‐CD44 axis. In conclusion, our study demonstrates that FAP+ CAFs are key players in orchestrating the CC microenvironment, and targeting the FAP‐driven immunosuppressive stem cell niche represents a promising strategy for future CC immunotherapy. Furthermore, considering our previous findings on the key regulatory role of FXR in intestinal stemness and inflammation during ulcerative colitis [31], the complex mechanisms governing stem cell dynamics and microenvironment remodeling across the colonic disease continuum warrant further investigation.

## Author Contributions

Qian Ding and Qingyan Lu designed the research. Qian Ding is responsible for data collection under the supervision of Qingyan Lu. Qingyan Lu conceived and planned the experiments and analyzed the data. Qian Ding wrote the first draft of the manuscript, and Qingyan Lu critically reviewed and revised the manuscript for important intellectual content, which was also improved by all authors. Qingyan Lu was responsible for the final content.

## Funding

This project is supported by the Science and Technology Development Fund of Affiliated Hospital of Xuzhou Medical University (Grant XYFM202343).

## Disclosure

The authors take full responsibility for the final content of the publication. All authors have read and approved the final manuscript.

## Conflicts of Interest

The authors declare no conflicts of interest.

## Data Availability

The data that support the findings of this study are available from the corresponding author upon reasonable request.
